# Cellular Localization of Aquaporin-1 in the Human and Mouse Trigeminal Systems

**DOI:** 10.1371/journal.pone.0046379

**Published:** 2012-09-28

**Authors:** Junying Gao, Meiyun Tan, Minxia Gu, Charles Marshall, Jiong Ding, Gang Hu, Ming Xiao

**Affiliations:** 1 Jiangsu Province Key Laboratory of Neurodegeneration, Department of Anatomy, Nanjing Medical University, Nanjing, Jiangsu, People's Republic of China; 2 Department of Rehabilitation Sciences, University of Kentucky Center For Excellence in Rural Health, Hazard, Kentucky, United States of America; Lerner Research Institute, United States of America

## Abstract

Previous studies reported that a subpopulation of mouse and rat trigeminal neurons express water channel aquaporin-1 (AQP1). In this study we make a comparative investigation of AQP1 localization in the human and mouse trigeminal systems. Immunohistochemistry and immunofluorescence results showed that AQP1 was localized to the cytoplasm and cell membrane of some medium and small-sized trigeminal neurons. Additionally, AQP1 was found in numerous peripheral trigeminal axons of humans and mice. In the central trigeminal root and brain stem, AQP1 was specifically expressed in astrocytes of humans, but was restricted to nerve fibers within the central trigeminal root and spinal trigeminal tract and nucleus in mice. Furthermore, AQP1 positive nerve fibers were present in the mucosal and submucosal layers of human and mouse oral tissues, but not in the muscular and subcutaneous layers. Fluorogold retrograde tracing demonstrated that AQP1 positive trigeminal neurons innervate the mucosa but not skin of cheek. These results reveal there are similarities and differences in the cellular localization of AQP1 between the human and mouse trigeminal systems. Selective expression of AQP1 in the trigeminal neurons innervating the oral mucosa indicates an involvement of AQP1 in oral sensory transduction.

## Introduction

Aquaporins (AQPs) function as water selective channels providing a major route for osmotically driven water transport through cell membranes [1]. To date, 13 mammalian AQPs have been characterized [2]. AQP1 is a 28 kD pore-forming membrane protein first discovered in human red blood cells [3]. Subsequent studies have revealed the presence of AQP1 in a variety of tissues and cells, including kidney tubules, microvascular endothelia, salivary glands and ciliary epithelia (For review, see Verkman, 2008) [4].

AQP1 is also expressed in the mammalian nervous system [5]. Within the brain, AQP1 is located in human and murine choroid plexus epithelia [6], [7], [8], mouse olfactory ensheathing glia [9] and human astrocytes [10], [11], [12]. In the peripheral nervous system, AQP1 is expressed in a subpopulation of primary sensory neurons of dorsal root and nodose ganglia in mice [13] plus enteric neurons in mice and rats [14], [15], [16]. Moreover, AQP1 mRNA or immunoreactivity has been observed in trigeminal ganglion neurons in rodents [13], [17], [18], [19], [20]. These results indicate that AQP1 could play a relevant role in trigeminal neurotransmission. However, the localization of AQP1 in the human trigeminal system and more detailed localization of this water channel in the rodent trigeminal system have not been investigated.

In the present study, we examined the cellular localization of AQP1 in the human and mouse trigeminal nerve systems by immunohistochemistry and immunofluorescence staining. Additionally, using fluorogold (FG) retrograde tracing combined with immunostaining, we identified a subpopulation of AQP1 positive trigeminal neurons that innervate the oral mucosa. These morphological evidences will contribute to the further exploration of the role of AQP1 in sensory functions and diseases associated with the trigeminal nerve.

## Results

### Expression and distribution of AQP1 in the human and mouse trigeminal ganglion

**Figure 1:**
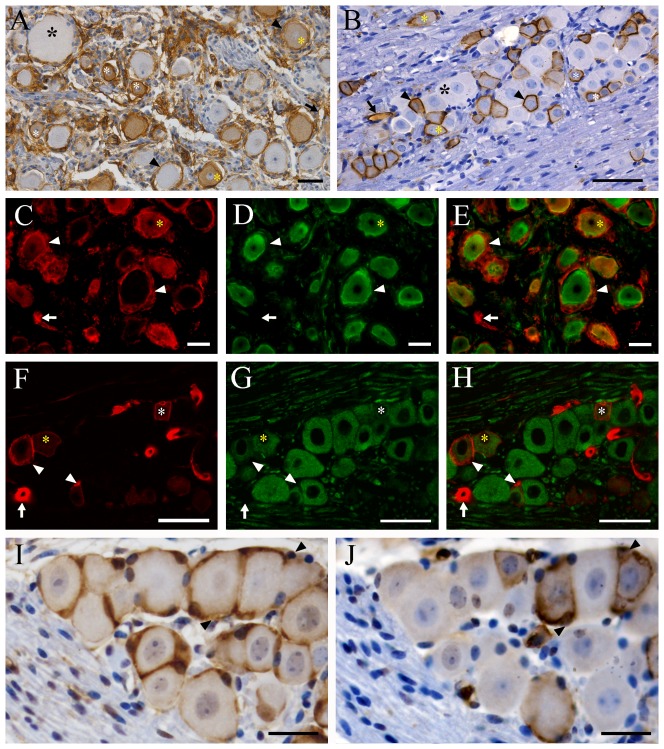
Localization of AQP1 in the trigeminal ganglion of humans (A, C–E) and mice (B, F–H). (**A–B)** Representative images of immunohistochemistry for AQP1. (**C–H)** Representative images of double immunofluorescence with AQP1 (red) and β-tubulin III (green). Some small-sized (white asterisks) and medium-sized (yellow asterisks) trigeminal neurons of humans and mice are positive for AQP1 which is localized to the cell membrane and cytoplasm. Large-sized trigeminal neurons (black asterisks) of the two species are negative for AQP1. Satellite cells expressing AQP1 (arrowheads) are observed around both AQP1-negative and -positive trigeminal neurons in humans, but only around AQP1-positive trigeminal neurons in mice. Both human and mouse capillary endothelial cells (arrows) express AQP1. (**I–J**) Immunolocalization of glutamine synthetase (I) and AQP1 (J) in the 10 µm adjacent sections of mouse trigeminal ganglion. Each neuron is wrapped tightly by GS-positive satellite glial cells (arrowheads in I). However, AQP1 immunoreaction (arrowheads in J) is only localized to satellite cells surrounding AQP1-positive trigeminal neurons. Scale bars = 50 µm in A–H; 15 µm in I and J.

Immunoreactivity for AQP1 was localized to the cytoplasm and cell membrane of medium and small-sized trigeminal neurons in humans and mice, as demonstrated by the immunohistochemistry (Figure 1A–B and Figure S1) and double immunofluorescence with β-tubulin III, a neuronal maker (Figure 1C–H). No AQP1 immunoreactivity was found in the large-sized trigeminal neurons in the two species.

**Figure 2:**
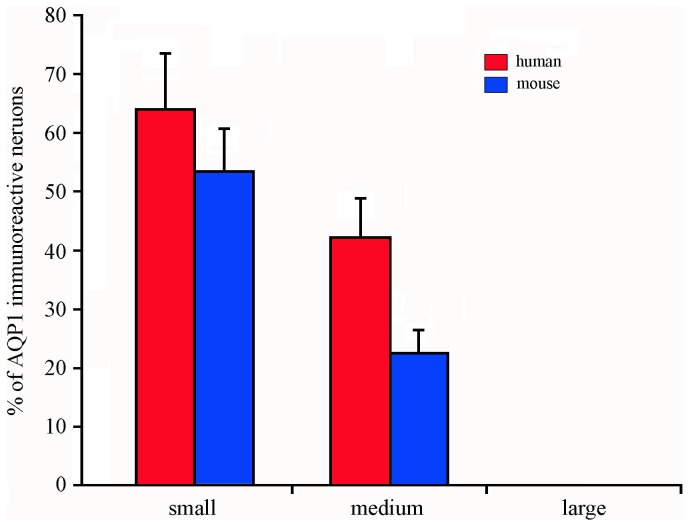
The percentage of AQP1-immunoreactive neurons in the trigeminal ganglia of humans and mice. The Data were expressed as a percentage of positive neurons respect to the total neuronal population in each class of neurons (small- medium- and large-sized neurons) (n = 3 in humans and n = 5 in mice).

The quantitative data showed that AQP1-immunoreactive neurons composed 39.74% in humans and 37.94% in mice of the total trigeminal neurons. Moreover, the percentages of small-sized and medium-sized trigeminal neurons immunoreactive for AQP1 were 63.65% and 43.7% in humans, and 53.28% and 22.47% in mice, respectively (Figure 2). The large-sized AQP1 positive trigeminal neurons were not observed in humans or mice.

All satellite cells were positive for AQP1 in human trigeminal ganglia (Figure 1A, C–E and Figure S1A–B), but only certain satellite glia which surround AQP1-positive trigeminal neurons expressed AQP1 in mice (Figure 1B, F–H and Figure S1C–D). In addition, AQP1 immunoreactivity was observed in the capillary endothelium of human and mouse trigeminal ganglia (Figure 1).

### Expression and distribution of AQP1 in the peripheral and central trigeminal branches of humans and mice

**Figure 3:**
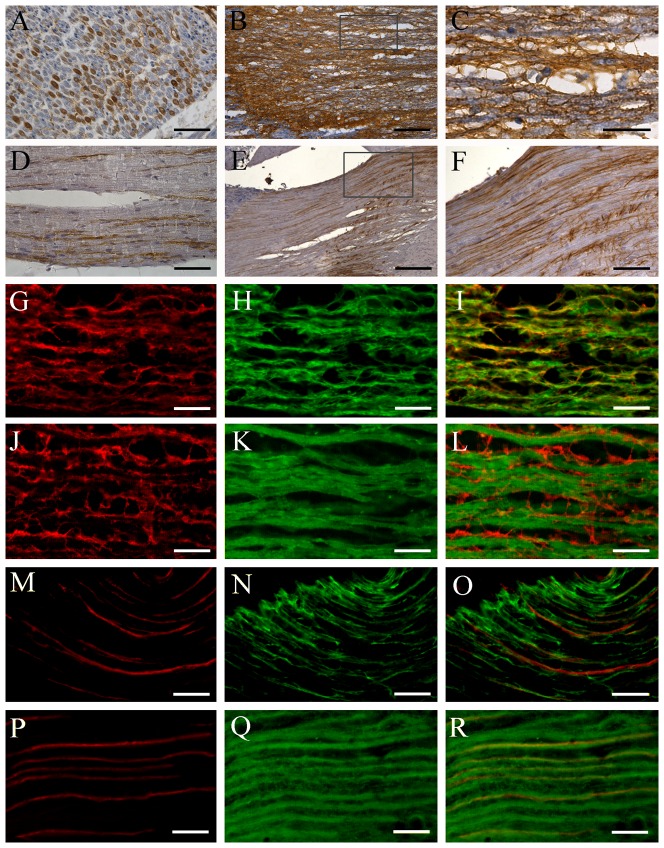
Localization of AQP1 in the peripheral and central branches of human (A–C,G–L) and mouse (D–F,M–S) trigeminal neurons. (**A–F**) Representative images of immunohistochemistry for AQP1. (**G–R**) Representative images of double immunofluorescence with AQP1 (red) and GFAP (green in H and N) or β-tubulin III (green in K and Q). A considerable proportion of axons are positive for AQP1 within the human (transverse section, A) and mouse (longitude section, D) mandibular nerve. The human trigeminal central root exhibits dense immunoreactivity for AQP1 (B, C, G, J), which is colocalized with GFAP (I), but not β-tubulin III (L). In contrast, in the mouse trigeminal central root, there are a population of AQP1 positive axons (E, F, M, P) that coexpress β-tubulin III (R) but not GFAP (O). Scale bars = 100 µm in A, D, F; 300 µm in B; 200 µm in E; 50 µm in C, G–R.

There were numerous AQP1 positive nerve fibers in the peripheral trigeminal branches in humans (Figure 3A) and mice (Figure 3D). In the central trigeminal root of humans, AQP1 was distinctly expressed in astrocytes (Figure 3B–C), which was further confirmed by the double immunofluorescence that AQP1 completely colocalized with astrocyte marker glial fibrillary acidic protein (GFAP) (Figure 3G–I) but did not overlap with neuronal maker β-tubulin III (Figure 3J–L). In contrast, in the central trigeminal root of mice, a considerable proportion of AQP1 positive nerve fibers were detected (Figure 3E–F) and colabeled for β-tubulin III (Figure 3P–R) but not for GFAP (Figure 3M–O).

### Expression and distribution of AQP1 in the human and mouse brain stem

**Figure 4:**
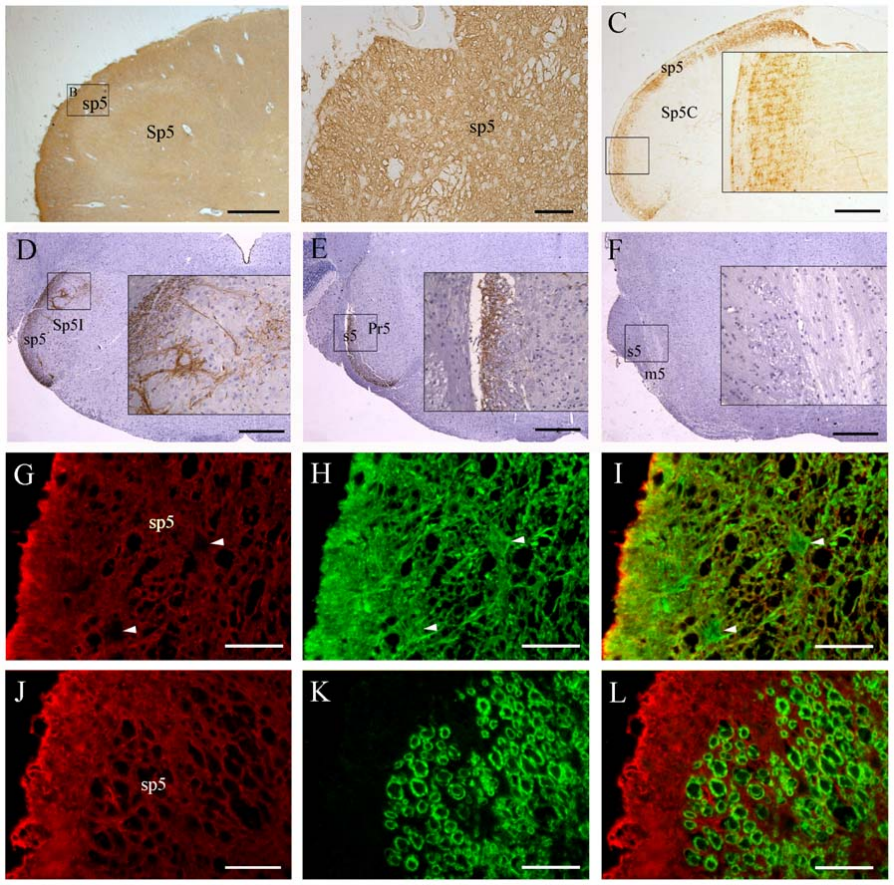
Localization of AQP1 in the brain stem of humans (A, B, G–L) and mice (C–F). (**A–F**) Representative images of immunohistochemistry for AQP1. (**G–L**) Representative images of double immunofluorescence of AQP1 (red) with GFAP (green in H) or β-tubulin III (green in K) in the sp5. There is extensive expression of AQP1 throughout the human medulla oblongata including the spinal trigeminal tract (sp5) and spinal trigeminal nucleus (Sp5, A and B). AQP1 and GFAP are highly colocalized at the glial lamellae along the medulla surface as well as astrocyte processes within the brain parenchyma (G–I). Interestingly, astrocyte cell bodies (arrowheads in I) do not express AQP1. No β-tubulin III positive axons coexpress AQP1 in the sp5 (L). In the mouse brain stem, dense dot-like AQP1 labeled nerve fibers are present in the caudal part of sensory root of the trigeminal nerve (s5, E) and sp5 (C and D), but not at the rostral part of s5 or at the motor root of the trigeminal nerve (m5, F). Moreover, AQP1 positive axonal terminals are observed in the caudal and interpolar parts of the trigeminal nucleus (Sp5C and Sp5I, C and D), but not in the principal sensory trigeminal nucleus (Pr5, E). Scale bars = 1 mm in A; 200 µm in B, G–L; 400 µm in C–F.

As shown in Figure 4A and B, AQP1 immunostaining was extensively distributed throughout the cross sections of the human medulla oblongata including the spinal trigeminal tract and the spinal trigeminal nucleus. Double immunofluorescence staining confirmed the colocalization of AQP1 with GFAP (Figure 4G–I) but not with β-tubulin III in the spinal trigeminal tract (Figure 4J–L). These results are consistent with the previous studies demonstrating that human astrocytes express AQP1 [10], [11], [12], [21]. In contrast, in the mouse brain stem, AQP1 labeled axons were specifically recognizable within the caudal part of the trigeminal root (Figure 4E), the spinal trigeminal tract and the spinal trigeminal nucleus (Figure 4C–D). AQP1 immunoreactivity was absent in the rostral part of the trigeminal root, motor root of the trigeminal nerve and principal sensory trigeminal nucleus (Figure 4E–F). Furthermore, the mesencephalic trigeminal nucleus and motor trigeminal nucleus also lacked immunoreactivity (data not shown). These results indicate that AQP1 is involved in conveying the superficial sensory information rather than conscious proprioception of the head.

### Expression and distribution of AQP1 in the human and mouse oral cavity tissues

**Figure 5:**
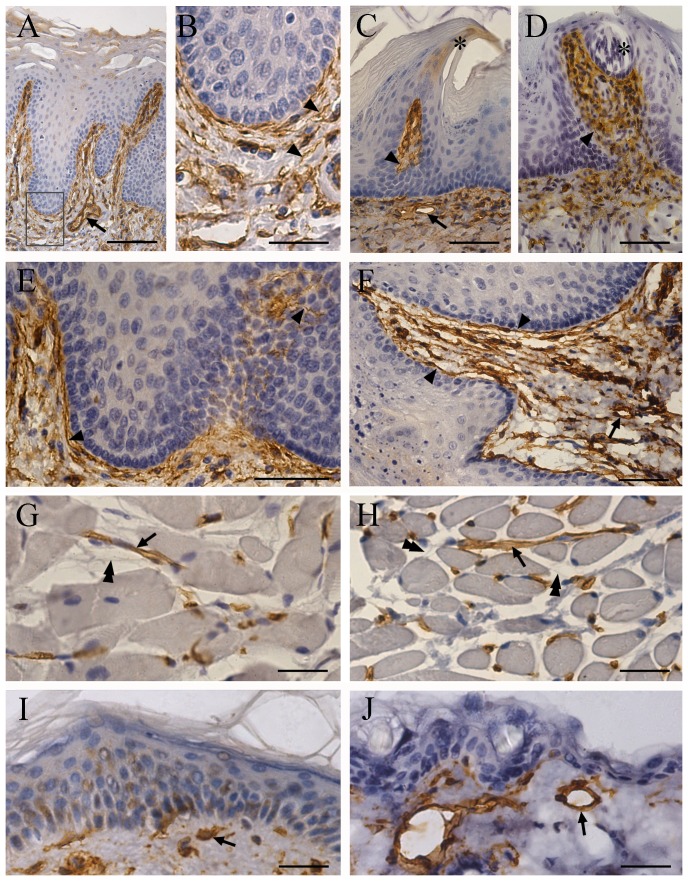
Localization of AQP1 in the oral tissues of humans (the left panels) and mice (the right panel). (**A–F**) Representative images showing that AQP1 is localized to nerve fibers (arrowheads in A–F) and microvascular walls (arrows in A–F) within the mucosa and submucosa of the tongue (A–D) and cheek (E and F) of the two species. There is AQP1 immunoreactivity within the filiform papillae of humans (A) and mice (B). Note that AQP1 immunoreactivity is distinctively localized to the apex of the mouse filiform papillae (stars in B). In addition, AQP1 immunoreactivity is found within the human stratum corneum of the tongue (A). AQP1 staining is present below the taste buds, but there is no staining within the taste buds (star in D). (**G–J**) Representative images showing that AQP1 immunoreactivity is present in blood vessels (arrows in G–J) in the human and mouse cheek muscle (G and H) and skin (I and J). Note that intramuscular nerve fibers (double arrowheads) are negative for AQP1. Scale bars = 200 µm in A; 50 µm in B and G–J; 100 µm in C–F.

Our results revealed that AQP1 immunoreactivity, aside from endothelial cells of capillaries and microvessels, was localized to nerve terminals within the mucosa and nerve fibers within the submucosa of tongue (Figure 5A–D), cheek (Figure 5E–F), palate and labium (data not shown) in both humans and mice. Interestingly, there was a considerable immunoreactivity of AQP1 in the stratum corneum of the oral mucosa in humans (Figure 5A). This expression pattern of AQP1 may assist the oral mucosa in rapidly absorbing water from the oval cavity. Moreover, AQP1 labeling was detected in the human and mouse tongue papillae (Figure 5A and C), but not in the taste buds (Figure 5D). No AQP1 immunopositive reactions were detected in the intramuscular or subcutaneous nerve fibers within the check of either species (Figure 5G–J). In addition, consistent with previous results [22], [23], AQP1 immunoreactivity was also detected in myoepithelial cells that form a basketlike framework around the acini and smaller ducts of the minor salivary glands within human and mouse oral tissues (data not shown).

**Figure 6:**
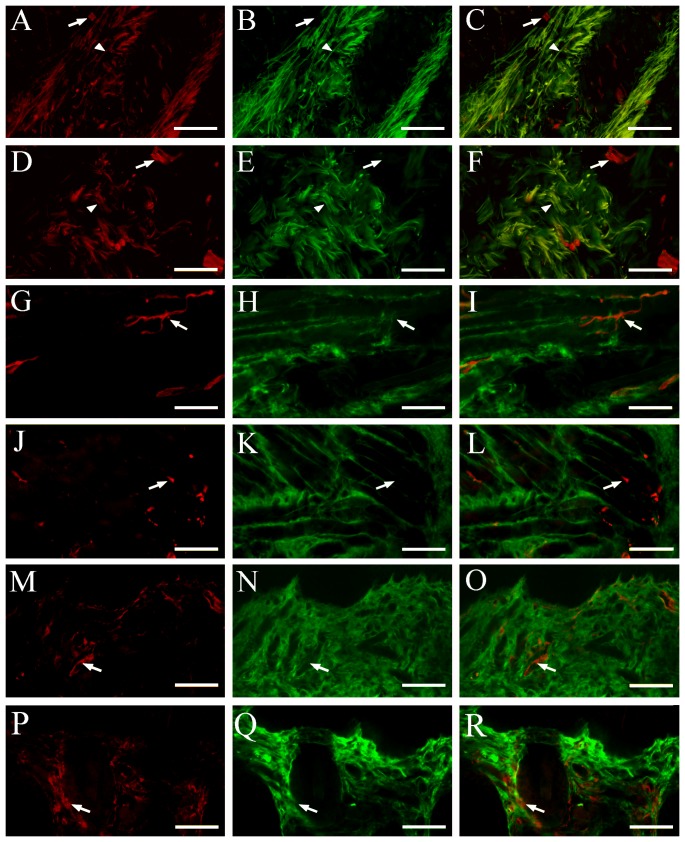
Colocalization of AQP1 and β-tubulin III in the oral submucosa, muscle and skin of humans (A–C, G–I, M–O) and mice (D–F, J–L, P–R). (**A–F**) AQP1 (red) and β-tubulin III (green) are coexpressed within a number of the nerve bundles (arrowheads) of the cheek submucosa from the two species. AQP1 positive microvessels (arrows) are also scatted among the nerve bundles. (**G–R**) There are a large number of β-tubulin III (green) positive nerve fibers, and a few AQP1 positive microvessels (arrowheads), within the cheek intermusclar (G–L) and subcutaneous regions (M–R). No overlap between β-tubulin III and AQP1 immunoreactivity is observed. Scale bars = 100 µm.

In order to confirm the selective innervation of AQP1-positive nerve fibers to the oral mucosa rather than skin or muscle, we performed the double immunofluorescence for AQP1 and β-tubulin III on the human and mouse check sections. As expected, a high colocalization of AQP1 and β-tubulin III was detected in a proportion of nerve fibers within the human and mouse cheek submucosa (Figure 6A–F). In contrast, no β-tubulin III positive nerve fibers coexpressed AQP1 in the check intramuscular regions (Figure 6G–L) or subcutaneous layers (Figure 6M–R). These results demonstrate a selective expression of AQP1 in the primary trigeminal afferent fibers innervating the oral mucosa.

### Mouse trigeminal neurons that innervate the oral mucosa but not skin express AQP1

**Figure 7:**
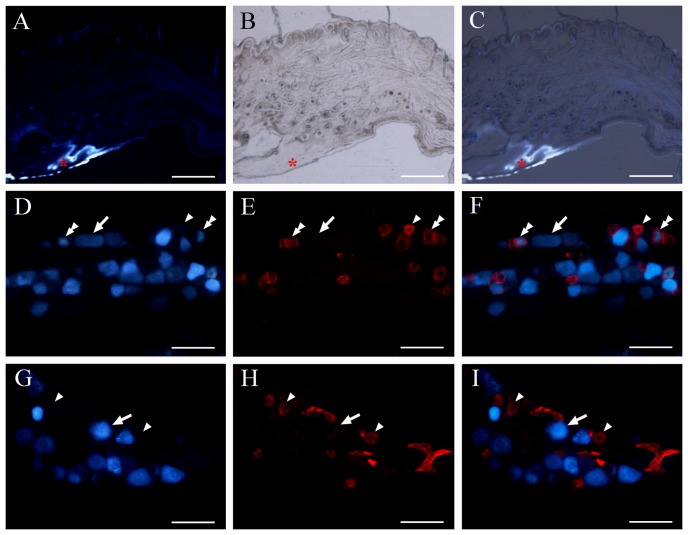
Mouse AQP1 positive trigeminal neurons innervate the oral mucosa but not skin. (**A–C**) Representative combined fluorescent and light images showing the injection site of retrograde tracer FG (red asterisks) within the cheek mucosa and submucosa of mice. (**D–I**) Representative double fluorescent images showing the co-distribution of AQP1 immunoreactive neurons (blue) with FG-labeled neurons (green) innervating cheek mucosa (D–F) or skin (G–I). AQP1 and FG double-labeled neurons are marked with arrowheads. AQP1 single-labeled neurons are marked with the double-arrowheads, and FG single-labeled neurons with arrows. Note that some FG-labeled small-size mucosal neurons are co-labeled with AQP1. No FG-labeled cutaneous neurons are positive for AQP1. Scale bars = 500 µm in A–C; 40 µm in D–I.

FG retrograde tracing, combined with immunofluorescence, was performed to confirm that AQP1 labeled nerve fibers in the submucosa were originated from the trigeminal sensory neurons (Figure 7A–C). A subset population of small to medium-sized trigeminal neurons co-labeled by FG and AQP1 were observed (Figure 7D–F). AQP1 immunofluorescent signals were absent within FG-labeled large-sized trigeminal neurons, which may be involved in proprioceptive pathway from the oral cavity to the brain [24]. Moreover, all FG-labeled neurons innervating the cheek skin were negative for AQP1 (Figure 7G–I). Together, these results further demonstrate that AQP1-positive trigeminal neurons specifically innervate the oral mucosa.

## Discussion

### Species specificity of AQP1 localization in the human and mouse trigeminal systems

In this study we compared the expression and distribution of AQP1 throughout the human and mouse trigeminal systems. The main results are summarized in Table 1. These results, for the first time, demonstrate the similarities and differences of AQP1 cellular localization in human and mouse trigeminal systems.

**Table 1:** Cellular localization of aquaporin-1 in the human and mouse trigeminal nervous system.

Regions	Neural elements	Humans	Mice
Trigeminal ganglion	Medium and small-sized neurons	+	+
	Large-sized neurons	−	−
	Satellite cells	+	+
Trigeminal peripheral root	Nerve fibers	+	+
Trigeminal central root	Astrocytes	+	−
	Nerve fibers	−	+
Brain stem	Astrocytes	+	−
	Nerve fibers within spinal trigeminal tract and nucleus	−	+
Oral tissues	Mucosal and submucosal nerve fibers	+	+
	Subcutaneous and intramuscular nerve fibers	−	−

+ represents AQP1 immunoreactivity is detectable and − represents is not.

Published data support the existence of species specificity of AQP1 localization in the mammalian nervous system, although the basis for these differences remains unclear. For example, earlier studies show that mouse and rat astrocytes express AQP4 but not AQP1 [25], [26], [27]. In contrast, several reports demonstrate that human astrocytes co-express AQP1 and AQP4 both *in vitro* and *in vivo* [10], [11], [12], [21]. In the peripheral nervous system, AQP1 has been observed in enteric neurons of rats [14] and sheep [28], but not humans [29]. The results from Shields and colleagues (2007) suggest that AQP1 is mainly expressed in a population of small-sized trigeminal ganglion neurons in mice [13]. On the other hand, Nandasena et al. (2007) reported that approximately two-thirds of AQP1 positive trigeminal neurons are medium-sized in rats [19]. These findings, together with the present results, suggest that the role of AQP1 for water transport and balance in the nervous system is essential but varied among different species.

### Selective expression patterns of AQP1 in the human and mouse trigeminal systems

The present results revealed several selective expression patterns of AQP1 in the human and mouse trigeminal systems. First, AQP1 is expressed in a subpopulation of small and medium sized trigeminal neurons, but not large sized neurons. Second, AQP1-positive nerve fibers are present in oral mucosa and submucosa, but not subcutaneous or intramuscular regions. Third, a subpopulation of small to medium-sized trigeminal neurons innervating mouse cheek mucosa, but not the skin, express AQP1. Finally, in the mouse brain stem, AQP1 positive axons are only observed in caudal part of the trigeminal root, the spinal trigeminal tract and nucleus.

Supporting the present results, selective expression of AQP1 in the nervous system is also found in the recent literature. For example, in the sheep duodenum, nearly 30% of submucosal neurons are immunoreactive for AQP1, whereas none of myenteric neurons are AQP1-positive [28]. Moreover, AQP1 is densely expressed in the mouse olfactory ensheathing glia located in the olfactory epithelium and olfactory bulb [9]. In contrast, no AQP1 is observed in mouse astrocytes of both the peripheral and central nervous systems [26], [30]. In addition, the present results reveal that AQP1 immunoreactivity is found in the peripheral, but not central, axons of human trigeminal ganglion neurons. In agreement with this, AQP4, another water channel protein in the nervous system, is strongly expressed in glial membranes that are in direct contact with capillaries and pia matter, thereby mediating water transport at the brain-blood and brain-cerebrospinal fluid interfaces [26], [30]. This polarized expression indicates that AQPs-mediated water movement exhibits a structural or regional specificity.

### Potential functions of AQP1 in the trigeminal system

It is well known that trigeminal neurons are responsible for transmission of orofacial somatosensory information. The selective expression of AQP1 in a subpopulation of small- and medium-sized trigeminal neurons innervating the oral mucosa indicates its potential role in oral somatosensory transduction. Several groups have addressed the roles of AQP1 in pain transmission in primary afferent neurons. An early study reported that AQP1 gene null mice exhibit mild impairment in nociceptive response after thermal (tail-flick) or chemical (capsaicin) stimuli [18]. In contrast, a subsequent study reported that, despite selective expression of AQP1 in nociceptive primary afferent of dorsal root ganglion neurons, there are no deficits in nociceptive processing in AQP1 null mice in a comprehensive battery of acute and persistent pain tests [13]. However, a recent study by Zhang and Verkman (2010) discovered a significant thermal inflammatory pain phenotype in AQP1 null mice evoked by bradykinin, prostaglandin E2, and capsaicin [31]. The study also found reduced cold pain perception in the mice, which was associated with action potential firing impairment in small dorsal root ganglion neurons.

There is still a lack of direct evidence for involvement of AQP1 in pain transmission of the trigeminal system. Borsani and colleagues (2009) investigated the expression of AQP1 and AQP2 in the trigeminal ganglia of mice, using an animal model of perioral acute inflammatory pain [20]. Their data have shown both increased and intracellular redistribution of AQP2 mainly in small-sized trigeminal neurons. Nevertheless, there were no alterations in AQP1 expression and distribution compared to the control group. This finding suggests that AQP2, but not AQP1, is involved in pain transmission of the trigeminal system. Further experiments are necessary to determine the contribution of AQP1 to trigeminal sensory transmission including nociceptive processing.

Apart from somatosensory transduction, we propose that expression of AQP1 in the trigeminal system may contribute to water sensation in the oral cavity. The moisture extent of the oral mucosa can be sensed by the central nervous system [32], however, there is little literature about osmosensory transduction from the oral cavity to the brain. A report by de Araujo et al. (2003) revealed that, by use of an event-related functional magnetic resonance imaging technique, the oral delivery of water activates the human frontal operculum/anterior insula and the caudal orbitofrontal cortex [33]. Moreover, the activations in the medial orbitofrontal cortex region triggered by water in the mouth are stronger when thirsty than when satiated. The distinctive expression of AQP1 in the subpopulation of trigeminal neurons innervating the oral mucosa may be a molecular basis for the brain responses to the stimulus of water from the oral cavity. More functional experiments, including investigations on AQP1 gene mice, are needed to prove this hypothesis.

In summary, our results demonstrate the similarities and differences in the cellular localization of AQP1 between the human and mouse trigeminal systems. We also provide the morphological evidence that AQP1 positive trigeminal neurons innervate the oral mucosa. These findings suggest that AQP1 may mediate trigeminal sensory transmission, but requires further study.

## Materials and Methods

### Human Specimens

Three cadavers without neurological or psychiatric illnesses, aged 36–70 years, were obtained via informed donation for the medical education and research of Nanjing Medical University with corresponding written consents prepared by the donors and their families. The utilization of human tissues was approved by the Ethics Committee of Nanjing Medical University.

### Animals

Sixteen three-month old male ICR mice were purchased from the Animal Breeding Facility of the Nanjing Medical University and housed under conditions of controlled illumination (12∶12 h light/dark cycle), humidity (30–50%), and temperature (18–22°C). Food and water were available at all times, except during the experiments. All procedures were approved by the Animal Welfare Committee of Nanjing Medical University. Every effort was made to reduce the number of animals and their suffering during the experiments.

### Microinjection of FG

For FG retrograde tracing experiments, mice were deeply anesthetized intraperitoneally using 3.5% chloral hydrate (350 mg/kg). One microlitre of 1% FG (Fluorochrom INC., Denver, CO, USA) in distilled water was injected into the cheek submucosa or subcutaneous layer (n = 5 in each group) via a 5-µl Hamilton microsyringe. After recovery from anesthesia, mice were returned to their home cages and sacrificed 3 days later.

### Tissue preparation

The cadavers were perfused through the internal carotid artery with 10,000 ml of a 10% formalin solution within 10 hours of death. The mice were deeply anesthetized with 2% sodium pentobarbital (40 mg/kg, i.p.), then transcardially perfused with 0.9% saline followed by 4% freshly-prepared paraformaldehyde in phosphate buffer (PB, 0.1 M, pH 7.4). Bilateral trigeminal ganglia of the two species were dissected and removed. The brain stem and oral tissues including the lip, soft and hard palates, cheek skin and mucosa, and tongue were also collected and each of them was cut into half. Human tissues were post-fixed in 4% paraformaldehyde/PB at 4°C for 3 days, and mouse tissues were post-fixed overnight.

One group of human and mouse tissues were cryoprotected overnight in 30% sucrose in 0.1 M PB, embedded in optimal cutting temperature compound (Fisher Scientific, Pittsburgh, PA, USA), and processed for frozen sections at 20–30 µm thickness using a cryostat (Leica CM1900, Leica, Nussloch, Germany). The other group was dehydrated in graded ethanol, embedded in paraffin and cut at a thickness of 6 µm using a paraffin slicing machine (Leica RM2135). Trigeminal ganglion samples were fixed in a horizontal position and cut into serial longitudinal sections. Samples of the mandibular division of the human trigeminal nerve, mouse and human oral tissues and brain stem were cut into transverse sections. All sections were mounted on gelatin-coated slides. In addition, the cheek and trigeminal ganglion tissues of FG injected mice were processed for frozen sections.

### Immunohistochemistry

Paraffin sections were deparaffinized with xylene and ethanol, followed by antigen retrieval with citric acid buffer (pH 6.0). After quenching endogenous peroxidase with 3% hydrogen peroxide for 10 min, the sections were incubated with blocking solution (0.01 M PBS containing 3% bovine serum albumin) for 1 hour at room temperature and overnight with the rabbit polyclonal anti-AQP1 (1∶1000, Millipore, Billerica, MA, USA) or anti- glutamine synthetase (GS) antibody (1∶200, Santa Cruz BioTech Santa Cruz, CA) at 4°C. After repeated washings in PBS, the sections were incubated with biotin-conjugated goat anti-rabbit IgG (1∶500) followed by horseradish peroxidase-conjugated avidin (ABC kit, Vector Laboratories, Burlingame, CA, USA). The positive reactions were visualized with 3,3′-diaminobenzidine tetrahydrochloride (DAB). Some sections were counter-stained with hematoxylin.

### Immunofluorescence

For double immunofluorescence staining, slide-mounted frozen sections were preincubated in 0.01 M PBS containing 0.2% Triton X-100 and 0.25% bovine serum albumin for 1 hour at room temperature. The sections were incubated overnight with a mixture of mouse monoclonal anti-β-tubulin III (1∶1000, Sigma-Aldrich, Saint Louis, MO, USA), or -GFAP antibody (1∶2000, Sigma-Aldrich) and polyclonal rabbit anti-AQP1 antibody (1∶1000, Millipore) overnight at 4°C. After rinsing, sections were incubated for 1 hour at room temperature in a mixture of fluorescein isothiocyanate-conjugated donkey anti-mouse IgG (1∶200, Vector Laboratories) and rhodamine isothiocyanate -conjugated donkey anti-rabbit IgG (1∶200, Vector Laboratories). The sections were then washed and cover-slipped with PBS/glycerol buffer.

For FG combined with AQP1 immunofluorescence staining, sections were preincubated in 0.25% bovine serum albumin PB for 1 hour at room temperature, and then incubated overnight with the polyclonal rabbit anti-AQP1 antibody (1∶1000, Millipore) at 4°C. After rinsing, sections were incubated with rhodamine isothiocyanate-conjugated donkey anti-rabbit IgG (1∶200, Vector Laboratories), then washed and cover-slipped with buffered PBS/glycerol.

To confirm the antibody specificity, we conducted immunostaining for AQP1 on human and mouse tissues where AQP1 protein has been described. In agreement with the current literature [4], [11], [12], we found that AQP1 was selectively and densely expressed at the apical surface of choroid plexus epithelium of humans and mice as well as the apical and basolateral membranes of mouse renal proximal tubules (Figure S2A–C).

In addition, the primary antibody for AQP1 was pre-absorbed with the corresponding antigen (Chemicon, Temecula, CA, USA) did not stain any specific immunoreaction in the above human and mouse tissues (Figure S2D–F). Negative controls were also performed by omitting the primary antibodies. All controls gave no detectable labeling.

### Image capture and analysis

The light or fluorescence micrographs were captured by Leica DM4000B digital microscope equipped with image capturing software (Leica Microsystems, Wetzlar, Germany). The cytoarchitecture of human and mouse brain stem sections with hematoxylin counterstaining was identified according to The Human Central Nervous System: A Synopsis and Atlas [34] and The Mouse Brain in Stereotaxic Coordinates [35], respectively. The FG injection sites were examined and captured by the blue channel. If the diffusion extent of FG was limited to submucosa or subcutaneous layer, sections of the trigeminal ganglion were processed for AQP1 immunofluorescence staining.

For quantitative analysis of AQP1 expression, paraffin sections of the human and mouse trigeminal ganglia, immunostained with AQP1 and counter-stained with hematoxylin, were captured in sequence at 400×magnification. Four sections containing the maximum plane of the trigeminal ganglia per specimen were selected for quantitative analysis. The cell number and cross-sectional areas of AQP1-positive and -negative trigeminal neurons with a clearly visible nucleolus were measured throughout the entire ganglion regions using an Image-Pro Plus 6.0 Analysis System (Media Cybernetics Inc., San Francisco, CA, USA). According to the previous studies [36], [37], [38], human and mouse trigeminal neurons were classified into the three types: small-sized neurons (the cross-sectional area <600 µm^2^ in humans and <300 µm^2^ in mice), medium-sized neurons (600–2000 µm^2^ in humans and 300–600 µm^2^ in mice) and large-sized neurons (>2000 µm^2^ in humans and >600 µm^2^ in mice). The percentages of AQP1-positive neurons with respect to the total number of neurons for each class were determined. Data are expressed as means ± standard derivation of the mean.

## Supporting Information

Figure S1 **AQP1 staining human and mouse trigeminal sections.** (**A–B**) Human satellite cells expressing AQP1 (arrowheads) are observed around either AQP1-negative neurons (stars) or AQP1-positive neurons (triangle). (**C–D**) In contrast, mouse AQP1-positive satellite cells (arrowheads) are only localized to AQP1-positive trigeminal neurons (triangle). No AQP1 immunoreactive signals are observed around AQP1-negative trigeminal neurons (stars). (**E–F**) After neutralizing rabbit-ant-AQP1 antibody by the C-terminal peptide, no immunostaining was present at the human (E) and mouse (F) trigeminal ganlia. Scale bars = 200 µm in A, 50 µm in B, 100 µm in C, 25 µm in D, 75 µm in E and F.
(TIF)

Figure S2 **Immunolocalization of AQP1 in human and mouse choroid plexus and mouse renal tissues.** (**A–C**) AQP1 immunoreactivity was selectively and densely expressed at the apical surface of human (A) and mouse (B) choroid epithelium and the apical and basolateral membranes of mouse renal proximal tubules (C). (**D–F**) Rabbit-ant-AQP1 antibody pre-incubated with the C-terminal peptide caused no immunostaining on the human (D) and mouse (E) choroid epithelium, and mouse renal tissues (F). Scale bars = 100 µm.
(TIF)
