# Supplementary figures and images for: Cellular Localization of Aquaporin-1 in the Human and Mouse Trigeminal Systems
Source: PLoS One. 2012 Sep 28;7(9):e46379. doi: 10.1371/journal.pone.0046379 (PMC3460814; doi:10.1371/journal.pone.0046379)

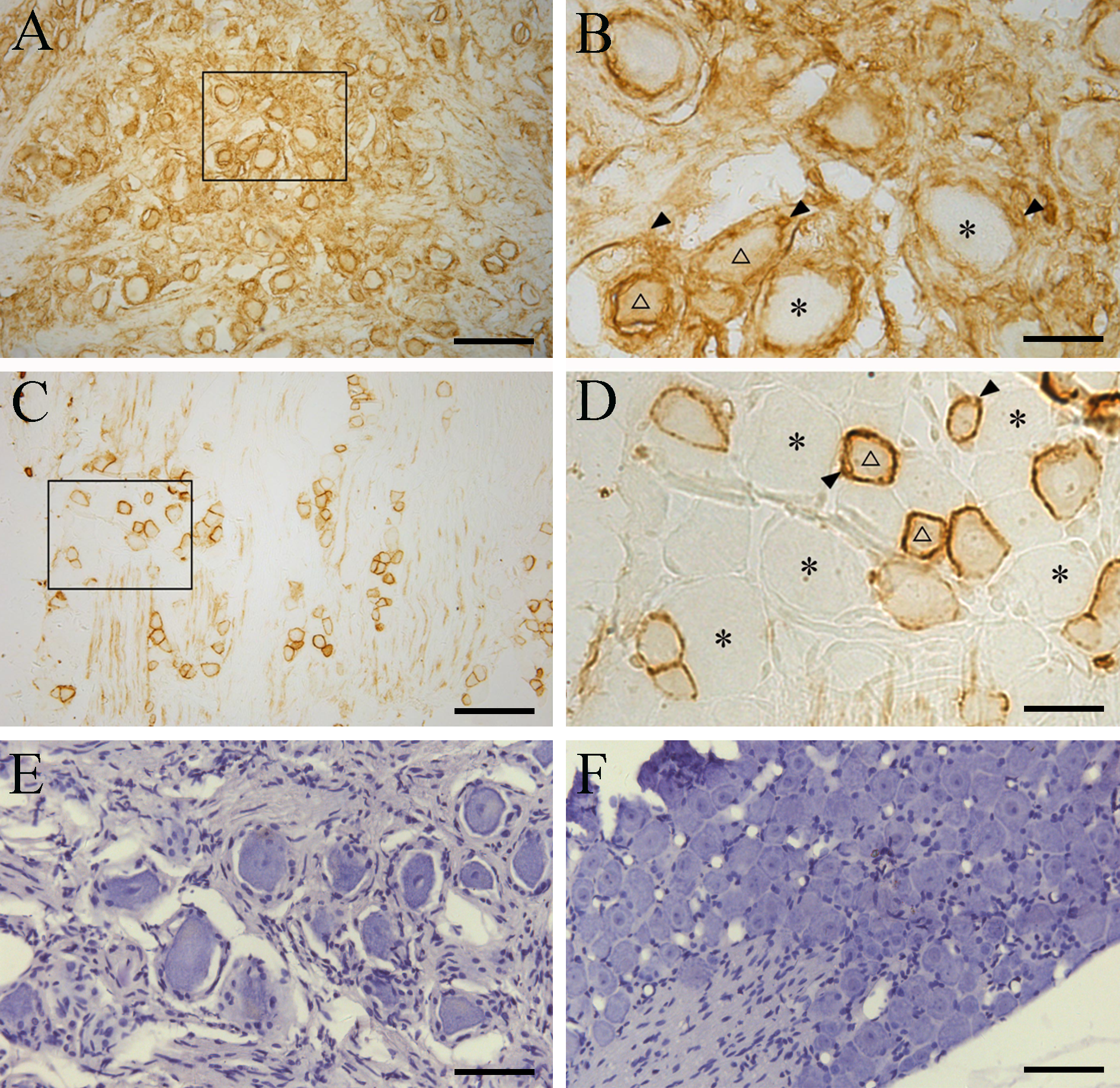

Supplement: Figure S1 — AQP1 staining human and mouse trigeminal sections. (A–B) Human satellite cells expressing AQP1 (arrowheads) are observed around either AQP1-negative neurons (stars) or AQP1-positive neurons (triangle). (C–D) In contrast, mouse AQP1-positive satellite cells (arrowheads) are only localized to AQP1-positive trigeminal neurons (triangle). No AQP1 immunoreactive signals are observed around AQP1-negative trigeminal neurons (stars). (E–F) After neutralizing rabbit-ant-AQP1 antibody by the C-terminal peptide, no immunostaining was present at the human (E) and mouse (F) trigeminal ganlia. Scale bars = 200 µm in A, 50 µm in B, 100 µm in C, 25 µm in D, 75 µm in E and F. (TIF) [file pone.0046379.s001.tif]

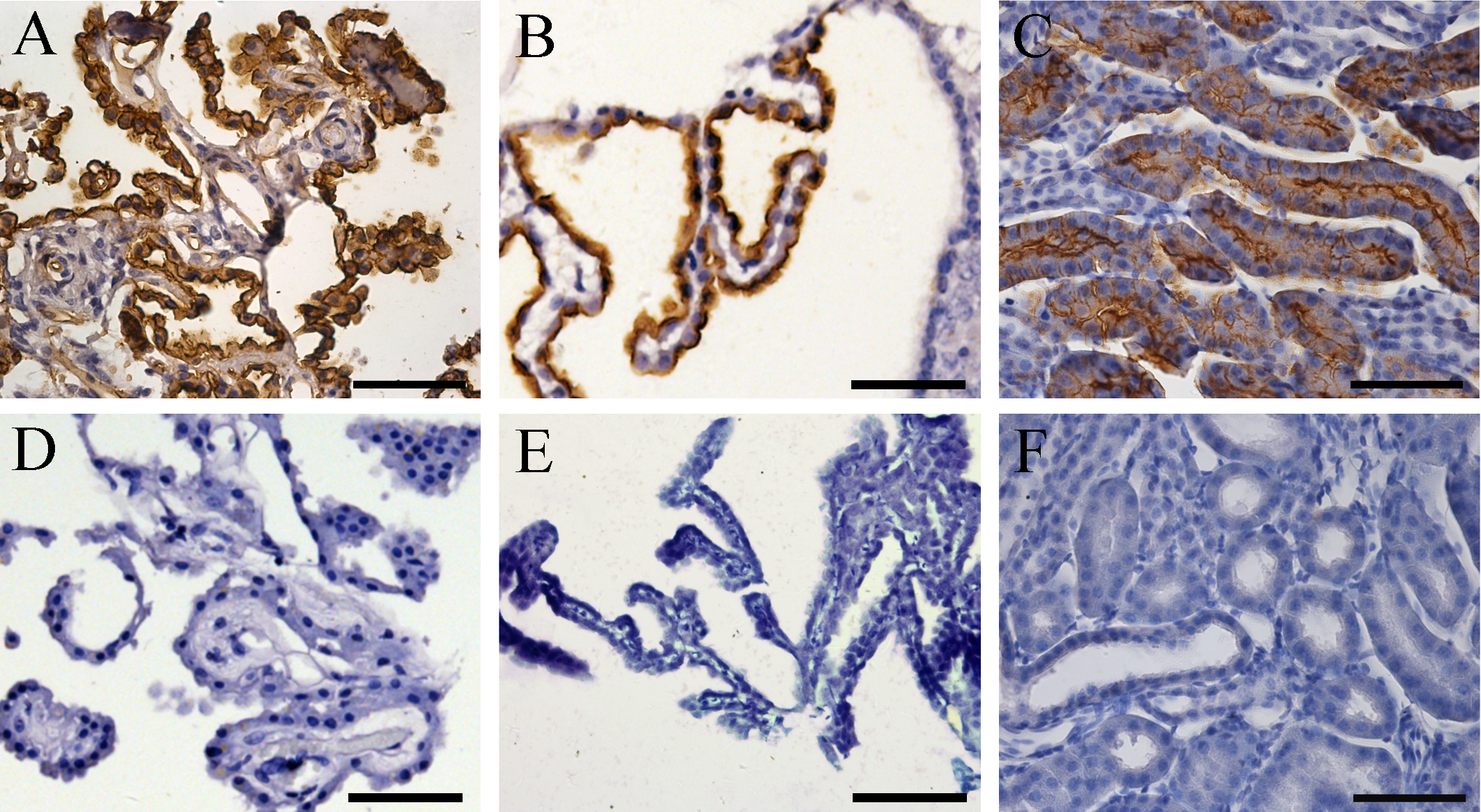

Supplement: Figure S2 — Immunolocalization of AQP1 in human and mouse choroid plexus and mouse renal tissues. (A–C) AQP1 immunoreactivity was selectively and densely expressed at the apical surface of human (A) and mouse (B) choroid epithelium and the apical and basolateral membranes of mouse renal proximal tubules (C). (D–F) Rabbit-ant-AQP1 antibody pre-incubated with the C-terminal peptide caused no immunostaining on the human (D) and mouse (E) choroid epithelium, and mouse renal tissues (F). Scale bars = 100 µm. (TIF) [file pone.0046379.s002.tif]
